# Paediatric Sjögren's Syndrome With Bilateral Parotid Cysts: A Case Report

**DOI:** 10.7759/cureus.43033

**Published:** 2023-08-06

**Authors:** Jamila Skinner, James Fowler, Jonathan Park, Peng You

**Affiliations:** 1 Department of Otolaryngology – Head and Neck Surgery, London Health Sciences Centre - Victoria Hospital, London, CAN; 2 Department of Pediatric Rheumatology, London Health Sciences Centre - Victoria Hospital, London, CAN

**Keywords:** otolaryngology, case report, paediatrics, parotid cysts, sjögren's syndrome

## Abstract

Sjögren's syndrome is an autoimmune disease characterized by the destruction of exocrine glands. Clinically, this results in the loss of tear and saliva production. Although xerophthalmia and xerostomia, also known as sicca, is a common presentation among adults, paediatric patients more often present with recurrent parotitis and glandular enlargement. Overall, symptoms can vary, making initial diagnosis challenging. Approximately 80% of patients with Sjögren's syndrome experience parotid gland enlargement, however, salivary cysts are rare. Herein, we present a case of paediatric Sjögren's syndrome where a 12-year-old female presented with a two-month history of bilateral parotid masses. The patient denied any history of xerostomia, xerophthalmia, or constitutional symptoms. Imaging revealed bilateral complex cystic intraparotid masses. A right parotid gland biopsy was performed showing parotid gland parenchyma with dense lymphoplasmacytic infiltrate. Ultimately, the presumptive diagnosis of Sjögren's syndrome was made. This case illustrates the importance of a thorough workup to aid in diagnostic certainty. Parotid cysts associated with Sjögren's are rare but should be considered within the differential diagnosis for paediatric patients with parotid swelling/mass.

## Introduction

Sjögren's syndrome is an autoimmune disease whereby the destruction of exocrine glands results in the loss of tear and saliva production [1]. The diagnostic criteria for Sjögren's syndrome are both complex and controversial [2]. Approximately 80% of patients with Sjögren's syndrome experience parotid gland enlargement, however, salivary cysts are rare [2]. Herein, we present a case of paediatric Sjögren's syndrome that presented as bilateral parotid cysts.

## Case presentation

**Figure 1:**
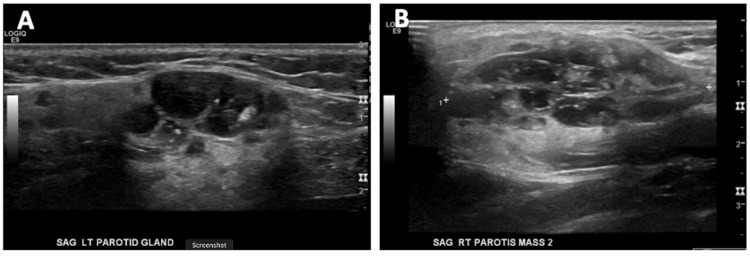
Sagittal ultrasound of the left (A) and right (B) parotid glands depicting bilateral complex solid/cystic intraparotid gland masses

**Figure 2:**
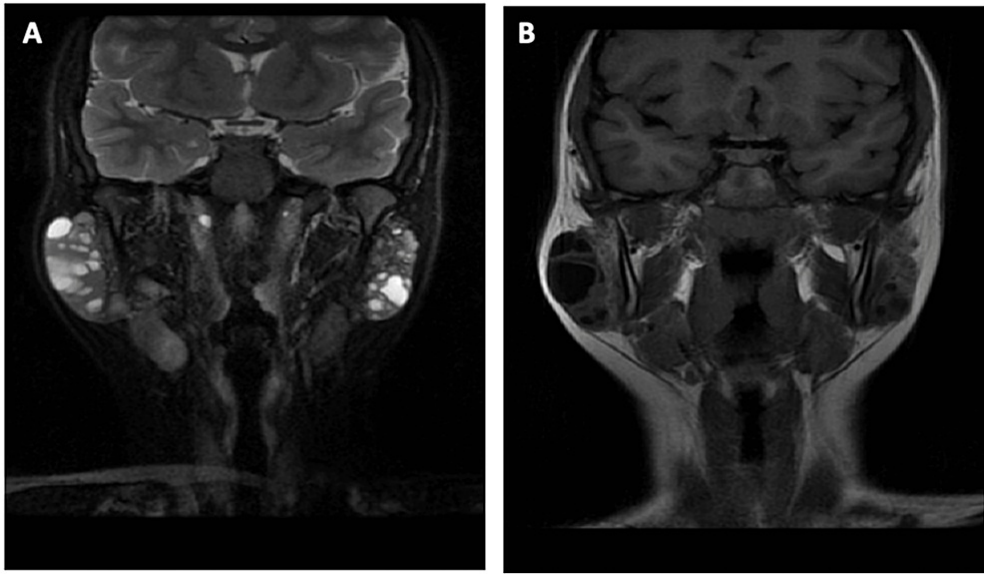
T2-weighted (A) and T1-weighted (B) magnetic resonance imaging head/neck demonstrate numerous cystic lesions in the parotid glands bilaterally Imaging criteria found no pathological lymph nodes.

A 12-year-old female presented with a two-month history of bilateral parotid masses. She reported no history of xerostomia, dry eyes, or constitutional symptoms. Ultrasound imaging revealed bilateral complex cystic intrapartoid masses measuring 4.7 x 3.1 x 1.7 cm on the right, with a second, more posterior and deep mass measuring 4.3 cm at its greatest diameter. The left mass measured 3.2 cm (Figure 1). Ultrasound imaging found the masses to be homogenous, with echogenic foci throughout. Magnetic resonance imaging confirmed numerous cystic lesions on the parotid bilaterally. These were hyperintense on T2, and hypointense on T1 (Figure 2).

Biopsy of the salivary glands is thought to be the single most specific test to confirm the diagnosis of Sjögren's syndrome [1]. As such, an ultrasound-guided core biopsy of the parotid gland was performed. Parotid gland parenchyma with dense lymphoplasmacytic infiltrate was found. The cyst was not aspirated. Pathologic findings from the core biopsy noted a lymphoplasmacytic infiltrate with the involvement of epithelial islands and reactive follicular hyperplasia.

Given the lack of diagnostic certainty, serology was ordered. Workup was negative for infectious aetiology such as human immunodeficiency virus, mumps, cytomegalovirus, and Epstein-Barr virus. Serology was positive for rheumatoid factor, as well as anti-Sjögren's-syndrome-related antigen A autoantibodies and anti-Sjögren's-syndrome-related antigen B consistent with a diagnosis of Sjögren’s syndrome.

The Sjögren Syndrome Foundation recommends treatment be prioritized as follows: 1) palliation of symptoms, 2) minimizing complications, and 3) proper patient selection when selecting immunosuppressive treatment [1]. Given that the patient was largely asymptomatic, ocular and oral symptoms did not need to be targeted directly. Instead, with the goal of decreasing inflammatory infiltrate in the parotids, the patient was treated with 200 mg of oral hydroxychloroquine daily. Theoretically, treatment with hydroxychloroquine reduces further cyst development by decreasing the active inflammatory infiltrate [1]. Further, hydroxychloroquine has a safe side-effect profile, lending itself to be a favourable treatment option for glandular presentations [1]. In severe cases, rituximab has been used to treat severe parotid gland swelling; however, its negative side-effect profile does not make it a first-line option [1]. In our case, the patient did not present with traditional symptoms, thus palliation of such symptoms with treatments such as artificial tears, punctal plugs or cauterization, and salivary stimulants were not initially considered. Treatments such as these could be initiated in the future should the patient's symptoms progress [1,2].

At two, five, and nine-month follow-ups, the patient has continued to tolerate the treatment well and has had no development of symptoms suggestive of active Sjögren's syndrome. It is not recommended to repeat biopsies to monitor disease activity; however, at the nine-month follow-up, a subjective decrease in parotid gland size was noted. This is possibly indicative of decreased inflammatory infiltrate. As in other systemic autoimmune diseases, prolonged and sustained remission on medication is often a good prognosticator. It is likely a reasonable goal to achieve several years of stable disease before considering discontinuing therapy. This is something that can be discussed with the patient and their family at future follow-up appointments and be guided by future data and the patient's continued response.

Further, due to the increased risk of developing lymphoma, the patient will continue to be screened for symptoms suggestive of lymphoma at each follow-up appointment. This includes a review of symptoms, a physical exam, routine bloodwork, and if findings are concerning, imaging. 

## Discussion

The occurrence of parotid cysts with Sjögren’s syndrome has been reported but is very rare, especially in the paediatric population [2]. The handful of cases that report bilateral parotid cysts as the presenting symptom of Sjögren’s syndrome echo the same challenges with the complex diagnostic criteria and further highlight the importance of a thorough workup in aiding in diagnostic certainty. The 2002 American-European Consensus Group Criteria for Sjögren’s syndrome is the most commonly used diagnostic tool (Table 1) [2]. Our case fulfils only criteria VI, which speaks to this diagnostic enigma.

**Table 1:** American-European Consensus Group criteria for Sjögren’s syndrome It requires four of the above six conditions to be met, one of which must include either IV or VI; alternatively, three of the four conditions must be met, including III, IV, V, and VI.

American-European Consensus Group Criteria for Sjögren’s Syndrome
I. Ocular symptoms (at least 1)
i. Dry eyes x 3 months
ii. Ocular foreign body sensation
iii. Use of artificial tears 3x per day
II. Oral symptoms (at least 1)
i. Dry mouth x 3 months
ii. Recurrent or persistently swollen salivary glands
iii. Need for liquids for swallowing dry foods
III. Ocular signs (at least 1)
i. Abnormal Schirmer’s test (<5 mm/5 min)
ii. Positive vital dye staining of the eye surface
IV. Histopathology
i. Biopsy showing focal lymphocytic sialadenitis
V. Oral signs (at least 1)
i. Unstimulated whole salivary flow (<= 15 mL/ 15 min)
ii. Abnormal parotid sialography
iii. Abnormal salivary scintigraphy
VI. Autoantibodies (at least 1)
i. Anti-Sjögren's syndrome-related antigen A autoantibodies (Ro)
ii. Anti–Sjögren's syndrome-related antigen B (La)

The present case suggests that, although rare, parotid cysts associated with Sjögren's syndrome should be considered within the differential diagnosis for any patient with parotid swelling or mass. A typical differential for parotid cysts includes mumps, human immunodeficiency virus, lymphatic malformations, cystic Warthin tumours, and branchial cysts [2]. These conditions can often be confirmed either clinically, with serology, or through imaging. Although, mumps does present with bilateral enlargement of the salivary glands - typically the parotids - it is usually diagnosed clinically due to its infectious nature [3]. Serology and specific histological changes are sufficient to confirm the diagnosis of human immunodeficiency virus [3]. Lymphatic malformations are commonly unilateral and would not present with systemic involvement [3]. The diagnosis of Warthin tumours relies on histopathological examination identifying cystic structures lined by oncocytic epithelial cells and lymphoid stroma with germinal centres [4]. Finally, the drainage tract of branchial cysts contributes to their identification [3].

Sjögren's syndrome is typically identifiable by its systemic clinical manifestations such as xerophthalmia, xerostomia and extraglandular manifestations [3]. When these symptoms are not present, it poses a diagnostic challenge. This lack of typical Sjögren's syndrome features in the presence of parotid cysts is what makes our case unique. Ahmad et al. describe a similar case where a paediatric patient presented without the typical features of Sjögren's syndrome [5]. Similarly, Ahmad et al. utilized imaging and cytology to confirm their diagnosis of Sjögren's syndrome [5]. While computed tomography and magnetic resonance imaging provide benefits in determining the extent of disease as well as identifying tumour lesions, the best confirmatory tests are biopsy and immunologic markers - specifically anti-Sjögren's-syndrome-related antigen A autoantibodies, being more sensitive and anti-Sjögren's-syndrome-related antigen B being more specific autoantibody tests [5]. Common pathological changes seen in Sjögren's syndrome include lymphocytic infiltration, destruction of the acini, and fibrosis [2]. While the specific pathogenesis of abnormal adipose deposition is unclear, there is a thought that it may be related to the destruction and shrinkage of the excretory system [2]. 

Of note, patients with Sjögren's syndrome are at a greater than 40% increased risk of developing lymphoma [1,2]. Not only must lymphoma be ruled out on the differential, but also long-term screening is essential for early detection, further emphasizing the importance of prompt and accurate diagnosis [2].

## Conclusions

The diagnosis of Sjögren's syndrome in paediatric patients can be complex and challenging. We present a unique case of Sjögren's syndrome with bilateral intraparotid cysts. This case illustrates the importance of a thorough workup to aid in diagnostic certainty. Parotid cysts associated with Sjögren's syndrome are rare but should be considered within the differential diagnosis for any patient with parotid swelling/mass. Particularly in young patients, an accurate and prompt diagnosis is important, as long-term screening is required due to the progression of the disease and the development of complications such as lymphoma.

## References

[1] Carsons SE, Patel BC (2022). Sjogren Syndrome. https://www.ncbi.nlm.nih.gov/books/NBK431049/.

[2] Seo BF, Ju RK, Kwok SK, Oh DY (2014). Unusual Sjögren's syndrome with bilateral parotid cysts. Arch Craniofac Surg.

[3] D'Arco F, Ugga L (2020). Computed tomography and magnetic resonance imaging in pediatric salivary gland diseases: a guide to the differential diagnosis. Pediatr Radiol.

[4] Limaiem F, Jain P (2023). Warthin Tumor. https://www.ncbi.nlm.nih.gov/books/NBK557640/.

[5] Ahmad I, Ray J, Cullen RJ, Shortridge RT (1998). Bilateral and multicystic major salivary gland disease: a rare presentation of primary Sjögren's syndrome. J Laryngol Otol.

